# Associação entre Terapia com Estatinas e Menor Incidência de Hiperglicemia em Pacientes Internados com Síndromes Coronarianas Agudas

**DOI:** 10.36660/abc.20200128

**Published:** 2021-02-19

**Authors:** Remo Holanda de Mendonça Furtado, Paulo Rizzo Genestreti, Talia F. Dalçóquio, Luciano Moreira Baracioli, Felipe Galego Lima, André Franci, Roberto R. C. V. Giraldez, Fernando R. Menezes, Aline Gehlen Ferrari, Viviane Moreira Lima, Cesar A. C. Pereira, Carlos Alberto Kenji Nakashima, Rocio Salsoso, Lucas Colombo Godoy, José C. Nicolau

**Affiliations:** 1 Hospital das ClinicasFaculdade de MedicinaUniversidade de São PauloSão PauloSPBrasilInstituto do Coração (InCor), Hospital das Clinicas HCFMUSP, Faculdade de Medicina, Universidade de São Paulo, São Paulo, SP - Brasil; 2 Hospital Israelita Albert EinsteinSão PauloSPBrasilHospital Israelita Albert Einstein, São Paulo, SP - Brasil; 3 University of TorontoRinggold standard institutionPeter Munk Cardiac Centre TorontoOntarioCanadáUniversity of Toronto Ringgold standard institution - Peter Munk Cardiac Centre Toronto, Ontario – Canadá

**Keywords:** Estatinas, Síndrome Coronariana Aguda, Infarto do Miocárdio, Glicemia, Inibidores de Hidroximetilglutril-CoA Redutases

## Abstract

**Fundamento:**

O maior risco de se desenvolver diabetes com o uso de estatinas é um desafio para a segurança do uso dessa classe de medicamentos em longo prazo. No entanto, poucos estudos analisaram essa questão durante síndromes coronarianas agudas (SCA).

**Objetivos:**

Investigar a associação entre início precoce da terapia com estatina e níveis de glicemia em pacientes admitidos com SCA.

**Métodos:**

Este foi um estudo retrospectivo de pacientes hospitalizados por SCA. Pacientes que nunca haviam usado estatinas foram incluídos e divididos segundo uso ou não de estatina nas primeiras 24 horas de internação. O desfecho primário foi a incidência de hiperglicemia na internação (definida como pico de glicemia > 200mg/dL). Modelos de regressão logística e modelos lineares multivariados foram usados para ajuste quanto a fatores de confusão e um modelo de pareamento por escore de propensão foi desenvolvido para comparações entre os dois grupos de interesses. Um valor de p menor que 0,05 foi considerado estatisticamente significativo.

**Resultados:**

Um total de 2357 pacientes foram incluídos, 1704 deles alocados no grupo que receberam estatinas e 653 no grupo que não receberam estatinas nas primeiras 24 horas de internação. Após os ajustes, uso de estatina nas primeiras 24 horas foi associado com uma menor incidência de hiperglicemia durante a internação (OR ajustado = 0,61, IC95% 0,46-0,80; p < 0,001) e menor necessidade de uso de insulina (OR ajustado = 0,56, IC 95% 0,41-0,76; p < 0,001). Essas associações mantiveram-se similares nos modelos de pareamento por escore de propensão, bem como após análises de sensibilidade, como exclusão de pacientes que desenvolveram choque cardiogênico, infecção grave ou pacientes que foram a óbito durante a internação hospitalar.

**Conclusões:**

Entre os pacientes internados com SCA que não receberam estatinas previamente, a terapia precoce com estatina associou-se independentemente com menor incidência de hiperglicemia durante a internação. (Arq Bras Cardiol. 2021; 116(2):285-294)

## Introdução

Há evidências bem estabelecidas de que as estatinas melhoram desfechos cardiovasculares em pacientes com doença arterial coronariana (DAC) estável.^1,2^ Ao mesmo tempo, pacientes com risco aumentado de DAC mas sem aterosclerose evidente podem se beneficiar do tratamento com estatinas,^3,4^ de modo que as diretrizes recomendam seu uso para esses dois grupos de pacientes.^5^ Ainda, as estatinas exercem importante papel nas síndromes coronarianas agudas (SCA)^6,7^ e, nos pacientes submetidos à revascularização percutânea, a terapia precoce pode prover benefício adicional.^8,9^ Apesar disso, existe a preocupação acerca do risco aumentado de se desenvolver diabetes mellitus (DM) com o uso prolongado de estatina.^10-12^ Ainda, as estatinas podem piorar o controle da glicemia em pacientes com diagnóstico de DM, ou antecipar a evolução para DM evidente em pacientes com síndrome metabólica, níveis alterados de glicemia de jejum ou intolerância à glicose.^13,14^ Muitos mecanismos foram propostos para explicar a influência de estatinas sobre a glicemia.^15^ As estatinas poderiam afetar o funcionamento de células beta e diminuir a secreção de insulina, um mecanismo diretamente relacionado à inibição de 3-hidroxi-3-metilglutaril-coenzima A (HMG-CoA) redutase ou a outros potenciais mecanismos intracelulares.^16,17^ Por outro lado, também há evidência de que as estatinas possam diminuir a resistência insulínica, o que compensaria o mecanismo danoso mencionado anteriormente.^18^

Embora existam muitos dados acerca da influência em longo prazo das estatinas sobre a glicemia em pacientes com DAC, dados sobre indivíduos com SCA são escassos. Apesar da preocupação mencionada com os efeitos sobre a tolerância à glicose em longo prazo, devido à ação anti-inflamatória das estatinas,^19,20^ estes medicamentos poderiam diminuir a inflamação na fase aguda das SCA e, portanto, indiretamente reduzir os níveis de glucose relacionados ao estresse da fase aguda. Assim, nossa hipótese é a de que, em pacientes internados com SCA, o uso precoce de estatina estaria associado a menor incidência de hiperglicemia durante a internação hospitalar na unidade coronariana (UCO).

## Métodos

### População e variáveis do estudo

Conduzimos uma análise retrospectiva de dados de pacientes admitidos com diagnóstico de SCA na UCO do Instituto do Coração da Faculdade de Medicina da Universidade de São Paulo. Todos os pacientes consecutivos admitidos em nossa UCO com diagnóstico de SCA foram prospectivamente registrados em um banco de dados específico, de 01 de janeiro de 1998 a 01 de maio de 2019. Nós identificamos pacientes que nunca haviam recebido estatinas na admissão hospitalar, e comparamos pacientes que receberam estatinas nas primeiras 24 horas de admissão (e continuaram o uso durante toda a internação) com pacientes que não as receberam.

Variáveis relacionadas ao tipo de SCA – infarto agudo do miocárdio com supradesnivelamento do segmento ST (IAMCSST), infarto agudo do miocárdio sem supradesnivelamento do segmento ST (IAMSSST), e angina instável (AI) – características demográficas basais, fatores de risco, história de DAC, procedimentos prévios, uso de medicamentos concomitantes, e resultados laboratoriais basais também foram coletados. A glicemia foi obtida por amostras diárias de sangue, e a primeira glicemia (obtida na admissão) e o valor mais alto de glicemia observado durante a internação (isto é, pico de glicemia) foram registrados no banco de dados para posterior análise.

Casos de SCA foram definidos como pacientes apresentando sintomas de isquemia em repouso ou piora de sintomas isquêmicos em esforço, que o levaram à internação urgente na UCO dentro dos primeiros sete dias do início dos sintomas. Infarto do miocárdio (IM) foi definido segundo a definição universal atual de IM durante a coleta de dados. IAMCSST foi definido como elevação persistente do segmento ST de pelo menos 1 mm em duas ou mais derivações contíguas (exceto em V2-V3, em que se definiu um aumento de pelo menos 1,5mm em homens e mulheres com idade maior que 40 anos e de pelo menos 2 mm em homens com idade menor que 40 anos) ou bloqueio do ramo esquerdo novo ou presumidamente novo no eletrocardiograma de admissão. Os casos que não atingiram os critérios para IM foram classificados como AI. Pacientes em uso de estatina imediatamente antes da internação-índice ou pacientes que não possuíam informações de níveis de glicemia ou terapia com estatina na internação-índice foram excluídos.

O desfecho de interesse primário em nossa análise foi a ocorrência de hiperglicemia durante a internação, definida como pico de glicemia > 200 mg/dL em qualquer momento durante internação, e os desfechos secundários foram glicemia > o valor mediano de pico de glicemia em nossa amostra e hiperglicemia que necessitasse terapia insulínica endovenosa. Esse ponto de corte de 200 mg/dL baseou-se nas mais recentes diretrizes considerando o nível alvo sugerido para controle de glicemia em pacientes com SCA.^21^ Também exploramos as associações entre hiperglicemia e mortalidade hospitalar.

### Rotina laboratorial

Todos os pacientes admitidos com SCA tiveram amostra de sangue coletada de uma veia do antebraço durante a admissão. O sangue foi centrifugado e enviado para o laboratório, onde os níveis de glicose foram determinados por um procedimento padrão. Para essa primeira amostra, não foi requerido jejum, uma vez que estávamos interessados no primeiro valor aleatório da glicemia, além de outros valores laboratoriais de rotina.

### Análise estatística

As variáveis categóricas foram comparadas pelo teste do qui-quadrado ou teste exato de Fisher, e foram descritas como números absolutos e porcentagens. As variáveis contínuas foram descritas como médias e desvios padrões ou mediana e intervalos interquartis (IIQ), e comparadas usando-se o teste t de Student (se distribuição normal) para duas amostras ou o teste de Mann-Whitney (distribuição não normal). O teste de Shapiro Wilk e análise visual de histogramas foram usados para avaliação de normalidade.

Especificamente, o teste de Mann-Whitney foi usado nas análises não ajustadas para comparar os desfechos contínuos de glicemia entre os dois grupos de interesse. Para as análises não ajustadas em relação aos desfechos binários (incidência de hiperglicemia e morte hospitalar), foram usados modelos de regressão logística univariada.

Para ajuste quanto aos fatores de confusão, foram usados modelos de regressão multivariados nas análises ajustadas. Níveis de glicemia com transformação logarítmica para a glicemia de admissão e pico de glicemia foram incluídos em um modelo de regressão linear múltiplo. A transformação foi realizada a fim de manter a premissa de normalidade dos resíduos no modelo. O modelo incluiu, como variáveis independentes, dados demográficos basais e variáveis relacionadas a comorbidades. Um procedimento de seleção *stepwise* foi usado para ajustar o modelo, com um valor limiar de 0,02 para remover covariáveis e de 0,05 para adicionar covariáveis ao modelo. Um modelo de regressão logística foi desenvolvido seguindo-se as mesmas etapas para avaliar hiperglicemia como uma variável categórica (considerando as três definições mencionadas) e morte hospitalar. Os modelos foram ajustados quanto às seguintes covariáveis: idade, raça, sexo, DM, hipertensão, hipercolesterolemia, tabagismo, insuficiência cardíaca (IC), IM prévio, intervenção coronária percutânea (ICP) prévia, cirurgia de revascularização miocárdica prévia (CABG), acidente vascular cerebral prévio, *clearance* de creatinina (ClCr) < 60 mL/min, fenótipo de SCA (IAMCSST versus IAMSSST ou AI), Classe II de Killip ou mais, anos de admissão (antes ou após o ano de 2010, o qual foi o ponto médio do período de tempo do banco de dados), cobertura do seguro saúde (privado *versus* público), e escore GRACE (registro global de eventos coronarianos agudos; do inglês *Global Registry of Acute Coronary Events*).^22^ Alguns pacientes não possuíam valores de hemoglobina glicada (HbA1c) e índice de massa corporal (IMC) disponíveis e não foram incluídos no modelo principal, mas foram incluídos em análises de sensibilidade (ver abaixo).

Além disso, um modelo de pareamento por escore de propensão foi desenvolvido considerando a probabilidade de se receber estatina nas primeiras 24 horas após admissão. O modelo foi construído a partir de regressão logística, com um vizinho mais próximo de 1, e nível de calibração (*caliper*) de 0,001, utilizando as mesmas variáveis usadas nos modelos de regressão. Após o pareamento, as variáveis basais foram conferidas entre os dois grupos para verificar eventual desequilíbrio, com valor de p maior que 0,10 e uma diferença padronizada menor que 10% considerados apropriados, segundo literatura prévia sobre o tópico.^23^

Para análise de sensibilidade, também aplicamos modelos ajustados para as variáveis basais, bem como para o uso de aspirina, inibidores de enzima conversora de angiotensina (IECA), bloqueadores de receptor da angiotensina II (BRA), betabloqueadores orais, inibidores de P2Y_12_, heparina não fracionada, heparina de baixo peso molecular, betabloqueadores endovenosos, nitratos e inibidores da glicoproteína IIbIIIa endovenosos nas primeiras 24 horas da admissão. Além disso, usamos também um modelo incluindo IMC (Kg/m^2^) e outro incluindo HbA1c na admissão hospitalar e covariáveis. Finalmente, também aplicamos modelos excluindo pacientes que apresentaram choque cardiogênico, infecção grave, ou que evoluíram a óbito durante a hospitalização.

Não foi empregada imputação de dados faltantes. Somente indivíduos com informações válidas sobre uso de estatina e níveis de glicemia foram incluídos. Todos os testes foram bicaudais. Um valor de p menor que 0,05 foi considerado estatisticamente significativo. As análises foram realizadas usando o programa Stata,^TM^ versão 15.1 (Statacorp, College Station, TX, EUA).

## Resultados

### Análises descritivas

**Figura 1:**
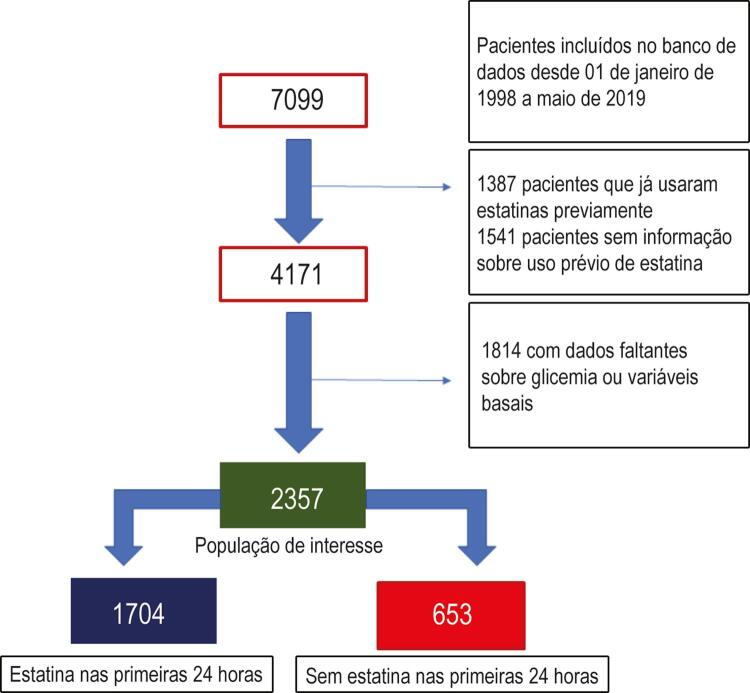
– Fluxograma representando o processo de inclusão e alocação dos pacientes.

Dos 7099 pacientes incluídos no banco de dados entre 01 de janeiro de 1998 e 01 de maio de 2019, 2357 pacientes que nunca usaram estatinas foram incluídos nesta análise, 1704 desses receberam estatina nas primeiras 24 horas da admissão e 653 não (Figura 1). Na população total do estudo, a idade média foi de 62,9 ± 12,6 anos, 713 pacientes (30,3%) eram do sexo feminino, 789 (33,5%) tinham história de DM na admissão, e 1073 (45,5%) IAMCSST como apresentação clínica das SCA (Tabela 1).

**Tabela 1:** – Características basais, valores laboratoriais, medicamentos nas primeiras 24 horas e estratégias de revascularização para o evento-índice de acordo com o grupo do estudo

Variáveis	Total (N = 2357)	Grupo com estatina (N=1704)	Grupo sem estatina (N=653)	Valor p
Raça branca	2102 (89,2)	1500 (88,0)	602 (92,2)	0,004
Sexo feminino	713 (30,3)	522 (30,6)	191 (29,3)	0,51
Idade (anos); média ± DP	62,9 ± 12,6	62,6 ± 12,5	63,8 ± 12,9	0,038
Diabetes	789 (33,5)	591 (34,7)	198 (30,3)	0,045
Hipertensão	1688 (71,6)	1246 (73,1)	442 (67,7)	0,009
Dislipidemia	1241 (52,7)	912 (53,5)	329 (50,4)	0,17
Tabagismo	611 (25,9)	450 (26,4)	161 (24,7)	0,39
IM prévio	653 (27,7)	461 (27,1)	192 (29,4)	0,25
CABG prévia	327 (13,9)	234 (13,7)	93 (14,2)	0,75
ICP prévia	413 (17,5)	304 (17,8)	109 (16,7)	0,51
AVC prévio	113 (4,8)	87 (5,1)	26 (4,0)	0,25
IC prévia	212 (9,0)	162 (9,5)	50 (7,7)	0,16
ClCr ≤ 60 mL/min	1316 (55,8)	1053 (61,8)	263 (40,3)	< 0,001
IAMCSST como evento-índice	1073 (45,5)	781 (45,8)	292 (44,7)	0,63
Classe II (ou mais) de Killip	447 (19,0)	308 (18,1)	139 (21,3)	0,075
Escore de GRACE, média ± DP	141,6 ± 47,5	140,5 ± 46,7	144,3 ± 49,4	0,14
Seguro de saúde público	1736 (73,7)	1334 (78,3)	402 (61,6)	< 0,001
Incluídos após janeiro 2010	950 (40,3)	870 (51,0)	80 (12,2)	< 0,001
IMC (kg/m^2^), mediana (IIQ)^1 ^	25,7 (23,4 – 28,7)	25,7 (23,3 – 28,6)	25,9 (25,0 – 29,1)	0,19
Colesterol total (mg/dL), mediana (IQR)^2^	182 (151-215)	182 (150-217)	178 (152-213)	0,51
LDL colesterol (mg/dL); mediana (IIQ)^2^	113 (87 – 143)	114 (86 – 144)	113 (90- 141)	0,96
Triglicerídeos (mg/dL); mediana (IIQ)^2^	128 (91-180)	129 (92-184)	122 (89-171)	0,042
HDL colesterol (mg/dL); mediana (IIQ)^2^	37 (31-44)	37 (31-44)	38 (31-45)	0,36
HbA1c (%); mediana (IIQ)^3^	5,9 (5,6 – 6,8)	5,9 (5,6 – 6,8)	5,9 (5,3 – 6,5)	0,054
Aspirina	2247 (95,4)	1646 (96,7)	601 (92,0)	< 0,001
Inibidor de P2Y_12_^4^	1199 (50,9)	1024 (60,1)	175 (26,8)	< 0,001
Betabloqueador oral	1424 (60,4)	1048 (61,5)	376 (57,6)	0,081
Betabloqueador endovenoso	189 (8,0)	95 (5,6)	94 (14,4)	< 0,001
Nitrato	1461 (62,0)	1002 (58,8)	459 (70,3)	< 0,001
HBPM	1326 (56,3)	1099 (64,5)	227 (34,8)	< 0,001
HNF	772 (32,8)	463 (27,2)	309 (47,3)	< 0,001
IECA/BRA	1651 (70,1)	1227 (72,0)	424 (64,9)	0,001
Inibidor da GpIIbIIIa	867 (36,8)	632 (37,1)	235 (36,0)	0,62
ICP primária	544 (23,1)	409 (24,0)	135 (20,7)	0,086
Fibrinolíticos	264 (11,2)	195 (11,4)	69 (10,6)	0,55
**Revascularização para o manejo do evento-índice^5^**				
ICP	1341 (56.9)	986 (57.9)	355 (54.5)	0.13
CABG	423 (18.0)	285 (16.7)	138 (21.1)	0.013
Abordagem clínica	637 (27.0)	460 (27.0)	177 (27.1)	0.96

Dados em número e % exceto se especificado de outra forma; 1- informação sobre índice de massa corporal (IMC) estava disponível em 287 pacientes; 2- informação sobre perfil de colesterol estava disponível de 2062 pacientes; 3- informação sobre hemoglobina glicada (HbA1c) estava disponível de 540 pacientes; 4- Sete pacientes, todos do grupo com estatina, estavam tomando ticagrelor nas primeiras 24 horas da internação, e todos os pacientes em terapia com inibidor de P2Y_12_ estavam em uso de clopidogrel; 5- o procedimento de revascularização cardíaca (CABG, coronary artery bypass grafting) e a intervenção coronária percutânea (ICP) não são necessariamente mutuamente exclusivas, uma vez que alguns pacientes possam ter sido submetidos a ambos. IECA: inibidor de enzima conversora de angiotensina II; BRA: bloqueadores de receptores da angiotensina 2; ClCr: clearance de creatinina; GpIIbIIIa: glicoproteína IIbIIIa; GRACE: Global Registry of Acute Coronary Events; IC: insuficiência cardíaca; IIQ: intervalo interquartil; HBPM: heparina de baixo peso molecular; HNF: heparina não fracionada; IM: infarto do miocárdio; IAMCSST: infarto agudo do miocárdio com supradesnivelamento do segmento ST.

Como o esperado, houve várias diferenças entre pacientes recebendo estatinas em comparação aos que não receberam estatinas nas primeiras 24 horas de internação. Os pacientes que receberam estatinas eram mais jovens, e mais propensos a apresentarem história de hipertensão, DM, e ClCr<60 mL/min na admissão, entre outras diferenças. Ainda, apresentavam maior chance de serem incluídos no banco de dados após janeiro de 2010. Contudo, esses pacientes apresentavam menos chance de serem da raça branca e possuírem seguro de saúde privado (Tabela 1). Os pacientes que receberam estatinas nas primeiras 24 horas também tinham maior probabilidade de serem tratados com aspirina, inibidor de P2Y_12_, e IECA ou BRA nas primeiras 24 horas (Tabela 1). Em relação aos valores laboratoriais, os pacientes recebendo estatinas nas primeiras 24 horas apresentaram níveis mais elevados de triglicerídeos e uma tendência de maiores níveis de HbA1c, mas níveis similares de colesterol total, LDL colesterol, e HDL colesterol (Tabela 1).

### Associação entre uso de estatina nas primeiras 24 horas e níveis de glicemia

Na análise não ajustada, os níveis de glicemia na admissão não foram diferentes entre pacientes que receberam estatinas e aqueles não recebendo estatinas nas primeiras 24 horas. No entanto, os pacientes tratados com estatinas apresentaram pico mais baixo de glicemia em comparação aos pacientes que não as receberam (Tabela 2A).

**Tabela 2A:** – Análise não ajustada 1: valores medianos da primeira medida de glicemia e do pico de glicemia de acordo com os grupos de estudo (com estatina ou sem estatina)

	Grupo com estatina (N=1704)	Grupo sem estatina (N=653)	Valor de p
Primeira medida de glicemia (mg/dL); mediana (IIQ)	116 (97 – 159)	113 (95 – 153)	0,22
Pico de glicemia (mg/dL); mediana (IIQ)	124 (101 – 175)	134 (106 – 196)	< 0,001

Na análise multivariada ajustada, a terapia com estatina nas primeiras 24 horas permaneceu independentemente associada com picos glicêmicos mais baixos na hospitalização (média geométrica ajustada de 139,0 versus 150,3 mg/dL, respectivamente; IC 95% da diferença de -15.9 a -6.5 mg/dL, p ajustado < 0,001). Após ajustes, não foram observadas diferenças significativas na glicemia de admissão (Tabela 2B).

**Tabela 2B:** – Análise multivariada 2: médias geométricas ajustadas da medida de glicemia de acordo com o grupo de estudo (com estatina e sem estatina)

	Grupo com estatina (N=1704)	Grupo sem estatina (N=653)	IC95% da diferença	Valor de p ajustado
Primeira medida de glicemia (mg/dL)	124.4	125.2	- 5.2 to 3.3	0.64
Pico de glicemia (mg/dL)	139.0	150.3	-15.9 to -6.5	< 0.001

Ajustado por idade, raça, sexo, diabetes mellitus, hipertensão, hipercolesterolemia, tabagismo, insuficiência cardíaca, infarto do miocárdio prévio, intervenção coronária percutânea prévia, revascularização do miocárdio prévio, clearance de creatinina < 60 ml/min, fenótipo de síndromes coronarianas agudas (IAMCSST versus IAMSSST), Classe II (ou mais) de Killip, escore GRACE, tipo de seguro saúde e período de inclusão (antes de janeiro 2010 versus depois de janeiro de 2010) 1- análise não ajustada pelo teste de Mann-Whitney. 2- análise multivariada realizada por regressão linear multivariada.

Na análise baseada no pareamento por escore de propensão, 500 pacientes do grupo de estatina foram pareados com um número similar de pacientes do grupo sem estatina. Após o pareamento, as características basais usadas para construir o modelo estavam bem balanceadas entre os dois grupos, sem nenhum valor de p inferior a 0,10, ou nenhuma diferença média padronizada maior que10% ( 1 Suplementar e Figura 1 Suplementar). Considerando a análise de pareamento por escore de propensão, a terapia com estatina permaneceu significativamente associada com níveis mais baixos de pico de glicemia (Tabela 2 Suplementar).

### Associação entre estatina e ocorrência de hiperglicemia durante internação

Na análise não ajustada, a terapia com estatina nas primeiras 24 horas foi associada com menor incidência de hiperglicemia, incluindo pico de glicemia acima de 200mg/dL, pico de glicemia acima da mediana, e hiperglicemia com necessidade de uso de insulina (Tabela 3 Suplementar).

**Tabela 3:** – Associações entre níveis de glicemia e morte hospitalar

	OR não ajustado (IC 95%); valor p	OR ajustado (IC 95%); valor p
Glicemia na admissão (para cada 10 mg/dL)	1,03 (1,01 – 1,06); < 0,001	1,02 (0,99 – 1,04); 0,12
Pico de glicemia (para cada 10 mg/dL)	1,06 (1,04 – 1,08); < 0,001	1,05 (1,03 – 1,07); < 0,001
Glicemia na admissão > 200 mg/dL	1,72 (1,15 – 2,56); 0,008	1,42 (0,90-2,24); 0,14
Pico de glicemia > 200 mg/dL	3,06 (2,20-4,28); <0,001	2,70 (1,76-4,16); <0,001

Ajustado por idade, raça, sexo, diabetes mellitus, hipertensão, hipercolesterolemia, tabagismo, insuficiência cardíaca, infarto do miocárdio prévio, intervenção coronária percutânea prévia, revascularização do miocárdio prévio, clearance de creatinina < 60 ml/min, fenótipo de síndromes coronarianas agudas (IAMCSST versus IAMSSST), Classe II (ou mais) de Killip, escore GRACE, tipo de seguro saúde e período de inclusão (antes de janeiro 2010 versus depois de janeiro de 2010). Modelos desenvolvidos usando regressão logística univariada para análises não ajustadas e regressão logística multivariada para análises ajustadas

**Figura 2:**
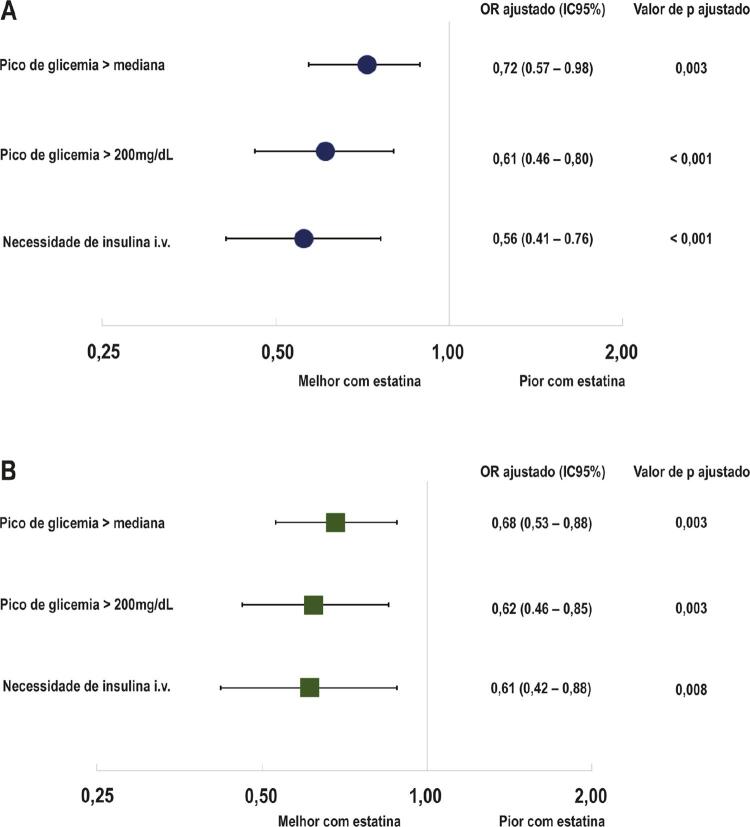
Risco ajustado de hiperglicemia durante internação segundo fierentes definições. A ajustado por regressão logística multivariada; B: ajustado por pareamento por escore de propensão Ajustado por idade, raça, sexo, diabetes mellitus, hipertensão, hipercolesterolemia, tabagismo, insuficiência cardíaca, infarto do miocárdio prévio, intervenção coronária percutânea prévia, revascularização do miocárdio prévio, clearance de creatinina < 60 ml/min, fenótipo de síndromes coronarianas agudas (IAMCSST versus IAMSSST), Classe II (ou mais) de Killip, escore GRACE, tipo de seguro saúde e período de inclusão (antes de janeiro 2010 versus depois de janeiro de 2010).

Após análise multivariada ajustada, a terapia com estatina permaneceu independentemente associada com menor incidência de pico de glicemia acima de 200 mg/dL (OR ajustado de 0,61, IC95% 0,46-0,80; p<0,001), e pico glicêmico acima da mediana e hiperglicemia com necessidade de terapia insulínica (ver Tabela 3 Suplementar e Figura 2A para mais detalhes).

Na análise de pareamento por escore de propensão, os resultados foram similares àqueles obtidos na regressão multivariada, com associações significativas entre uso de estatina e risco mais baixo de hiperglicemia de acordo com as três definições (Tabela 3 Suplementar e Figura 2B).

### Análises de sensibilidade

As associações entre a terapia com estatina e menor incidência de hiperglicemia (pico de glicemia > 200mg/dL) permaneceram consistentes mesmo após várias análises de sensibilidade, como por exemplo incluindo-se outras medicações concomitantes no modelo. Os resultados para o desfecho primário também mantiveram-se consistentes em um modelo que considerou a data de inclusão no banco de dados como uma variável contínua. Em outra análise, onde os pacientes foram estratificados de acordo com o período de inclusão (antes *versus* após janeiro de 2010), não houve modificação de efeito significativo para o desfecho primário. Além disso, quando os pacientes que desenvolveram choque cardiogênico ou infecção grave, ou que não sobreviveram até a alta hospitalar foram excluídos da análise, não restaram associações significativas entre o uso de estatinas nas primeiras 24 horas e a menor ocorrência de hiperglicemia. Finalmente, nos modelos que incluíram HbA1c (%) ou IMC como covariáveis, apesar de a estimativa-ponto para as OR ter sido similar, não restaram associações significativas, provavelmente devido ao pequeno número de pacientes com aquelas duas variáveis disponíveis. Esses resultados estão descritos nas Tabelas Suplementares 4-11.

### Associação entre níveis de glicemia e mortalidade hospitalar

Na população do estudo, observou-se uma associação independente entre valores mais altos de pico de glicemia e maior mortalidade hospitalar (OR 1,05; IC95% 1,03-1,07 para cada 10 mg/dL; p < 0,001). Por outro lado, a glicemia de admissão não se associou independentemente com mortalidade hospitalar (Tabela 3).

## Discussão

### Principais achados do estudo

Obtivemos resultados importantes neste estudo. Primeiramente, o uso de estatina nas primeiras 24 horas de internação hospitalar nas SCA foi altamente associado a comorbidades e, mais importante, observou-se uma associação temporal relevante, de modo que, em nossos dados, observou-se diferença importante entre pacientes incluídos antes de 2010 versus pacientes incluídos após 2010. Tal fato está provavelmente relacionado às evidências acumuladas sobre o uso de estatinas nas SCA,^6,7^ apesar de não existir nenhuma recomendação para se iniciar a terapia com estatina nas primeiras 24 horas de admissão.^5,21^ Segundo, houve uma associação independente entre o uso precoce de estatina e uma incidência mais baixa de hiperglicemia hospitalar. Essa associação foi observada em dois diferentes modelos ajustados (regressão logística e pareamento por escore de propensão) e após várias análises de sensibilidade realizadas para verificar a consistência dos achados. Portanto, apesar do risco de se desenvolver nova DM na fase crônica, nossos resultados provavelmente excluem qualquer prejuízo aparente à tolerância à glicose causado pelas estatinas durante a fase aguda de SCA, um período em que o aumento de catecolaminas e mediadores inflamatórios podem aumentar a susceptibilidade à hiperglicemia de estresse e suas potenciais consequências clínicas.^24^

### Comparação com estudos prévios

Apesar da vasta literatura investigando os efeitos crônicos das estatinas sobre a tolerância à glicose, poucos estudos investigaram qualquer efeito possível no cenário agudo. Yan et al.,^25^ relataram um risco aumentado de hiperglicemia de estresse em pacientes com IM agudo recebendo estatinas. Mas a ausência de análises ajustadas e a definição arbitrária do ponto de corte para hiperglicemia de estresse enfraquecem as conclusões daquele estudo. Sposito et al.,^26^ estudaram essa questão em pacientes hospitalizados com IAMCSST, e mostraram que 80mg de sinvastatina diminuiu a sensibilidade à insulina em comparação à 10 mg de sinvastatina avaliada pelo método do clamp euglicêmico hiperinsulinêmico.^26^ Apesar desses resultados parecerem contrastar com os nossos, a inclusão de somente pacientes sem DM e o uso do método do clamp euglicêmico hiperinsulinêmico limitam a generalização de seus resultados para um cenário real como é o caso do nosso estudo. No entanto, é possível que, apesar do efeito adverso sobre a resistência à insulina precocemente na fase aguda das SCA, as estatinas poderiam compensar tal efeito pela redução da resposta inflamatória, levando a uma diminuição nos níveis de glicemia.

A associação entre estatinas e glicemia mais baixa em situações de estresse poderia ser atribuída, ao menos em parte, pelos efeitos diretos das estatinas sobre inflamação, o que está bem estabelecido na literatura.^19^ Outros estudos sugeriram que aqueles efeitos pleiotrópicos das estatinas possam ocorrer precocemente nas SCA. Dois estudos randomizados demonstraram que o tratamento de curta duração (menos que 5 dias) com rosuvastatina, comparado a placebo, reduziu a incidência da lesão renal aguda pós-contraste.^27,28^ Esse efeito parece ser mediado por uma ação anti-inflamatória, uma vez que nenhum efeito hipolipemiante seria esperado em um prazo tão curto.^29^ Ainda, uma metanálise mostrou que uma dose de ataque precoce de estatina diminui a incidência de IM relacionado ao procedimento.^8^ Contudo, um ensaio randomizado mais amplo, o SECURE-PCI, não detectou uma redução em eventos isquêmicos pós SCA com atorvastatina precoce na dose de 80 mg,^9^ apesar do potencial benefício no subgrupo de pacientes submetidos à ICP após a randomização.^30^

### Hiperglicemia e mortalidade após SCA

O impacto da hiperglicemia sobre a sobrevida após SCA está bem estabelecido tanto em diabéticos como em pacientes sem DM.^24^ Em uma análise no estudo CARDINAL, Goyal et al.,^31^ sugeriram que a persistência de níveis elevados de glicose nas 24 horas após a admissão estava ainda mais associada com menor sobrevida que níveis elevados na admissão.^31^ Embora exista uma associação entre glicemia e mortalidade, ainda não se sabe se a hiperglicemia é um mediador direto de morte celular aumentada e lesão durante o IM, ou somente um marcador de risco basal aumentado. De uma perspectiva biológica, a hiperglicemia possivelmente está associada com danos diretos na microcirculação e remodelamento ventricular esquerdo.^32^ Por outro lado, resultados de estudos randomizados que não conseguiram demonstrar um melhor prognóstico com um controle mais rígido da glicemia apoiam a segunda hipótese.^33,34^ Apesar disso, se a hiperglicemia estiver de fato parcialmente implicada no dano miocárdico no IM, nossos resultados são tranquilizadores, uma vez que eles provavelmente excluem um efeito deletério das estatinas sobre o metabolismo glicêmico durante a fase aguda das SCA.

### Limitações do estudo

Nosso estudo possui várias limitações. Primeiramente, não coletamos informações detalhadas sobre dosagens e tipos de estatinas usadas. Enquanto alguns estudos mostraram distintos efeitos de diferentes estatinas sobre o metabolismo da glicose, outros sugeriram que o risco de DM com estatinas pode ser um efeito de classe.^35,36^ Segundo, nosso banco de dados abrange um longo período de tempo, incluindo pacientes desde 1998, ano em que o uso de estatinas na fase aguda do IM era menor. Porém, nós consideramos essa covariável nos modelos ajustados e em análises de sensibilidade, reforçando que a associação encontrada não foi enganosamente causada por esse fator de confusão. Terceiro, nós não coletamos informações detalhadas sobre indicações e contraindicações de se iniciar ou não terapia com estatina precocemente nas SCA. Assim, é possível que a chance de pacientes com maior risco de morte ou em condição clínica crítica receber estatinas nas primeiras 24 horas pelo médico tenha sido menor. Vários fatores, além do efeito do medicamento sobre o metabolismo da glicose, poderiam ter influenciado na decisão de se iniciar ou não estatinas nas primeiras 24 horas. Ainda, apesar de ajustes para várias comorbidades e outros fatores demográficos e socioeconômicos tenham sido realizados, fatores de confusão residuais desconhecidos podem ter permanecido. Entretanto, o escore GRACE, um preditor bem estabelecido de mortalidade hospitalar em pacientes com SCA^22^ foi incluído como covariável, e nós também conduzimos análises de sensibilidade excluindo pacientes que desenvolveram choque cardiogênico ou infecção grave, e pacientes que foram a óbito durante a primeira internação. Quarto, devido à natureza retrospectiva de nossa análise, não foi possível determinar se nossos resultados estiveram sujeitos a viés de recordação. Quinto, pelo fato de nossos dados serem derivados de um banco de dados de um único centro, não está claro se nossos resultados podem ser extrapolados para outros países ou para a realidade de outros hospitais. Finalmente, dada a natureza observacional do estudo, não podemos fazer nenhuma inferência causal, mas apenas concluir sobre associações, de modo que nossos achados são somente geradores de hipóteses e devem ser confirmados em ensaios randomizados específicos.

## Conclusão

Em pacientes admitidos com SCA, a terapia com estatina nas primeiras 24 horas associou-se com menor incidência de hiperglicemia durante internação hospitalar. Esse resultado sugere que, embora as estatinas possam aumentar o risco de novo DM em longo prazo, esses medicamentos podem estar associados a efeitos benéficos ao metabolismo da glicose em curto prazo nas SCA.
