# Supplementary material for: Associação entre Terapia com Estatinas e Menor Incidência de Hiperglicemia em Pacientes Internados com Síndromes Coronarianas Agudas
Source: Arq Bras Cardiol. 2021 Feb 19;116(2):285–94. [Article in Portuguese] doi: 10.36660/abc.20200128 (PMC7909967; doi:10.36660/abc.20200128)
Supplement: Translation

# Translation: Association between Statin Therapy and Lower Incidence of Hyperglycemia in Patients Hospitalized with Acute Coronary Syndromes

**Authors:** Remo Holanda de Mendonça Furtado, Paulo Rizzo Genestreti, Talia F. Dalçóquio, Luciano Moreira Baracioli, Felipe Galego Lima, André Franci, Roberto R. C. V. Giraldez, Fernando R. Menezes, Aline Gehlen Ferrari, Viviane Moreira Lima, Cesar A. C. Pereira, Carlos Alberto Kenji Nakashima, Rocio Salsoso, Lucas Colombo Godoy, José C. Nicolau

**Affiliations:** 1 BrasilInstituto do Coração (InCor), Hospital das Clinicas HCFMUSP, Faculdade de Medicina, Universidade de São Paulo, São Paulo, SP - Brasil; 2 BrasilHospital Israelita Albert Einstein, São Paulo, SP - Brasil; 3 CanadáUniversity of Toronto Ringgold standard institution - Peter Munk Cardiac Centre Toronto, Ontario – Canadá

**Keywords:** Statins, Acute Coronary Syndrome, Myocardial Infarction, Blood Glucose, Hidroxymethylglutaryl-CoA-Reductase Inhibition

## Abstract

**Background:**

Increased risk of new-onset diabetes with statins challenges the long-term safety of this drug class. However, few reports have analyzed this issue during acute coronary syndromes (ACS).

**Objective:**

To explore the association between early initiation of statin therapy and blood glucose levels in patients admitted with ACS.

**Methods:**

This was a retrospective analysis of patients hospitalized with ACS. Statin-naïve patients were included and divided according to their use or not of statins within the first 24 hours of hospitalization. The primary endpoint was incidence of in-hospital hyperglycemia (defined as peak blood glucose > 200 mg/dL). Multivariable linear and logistic regression models were used to adjust for confounders, and a propensity-score matching model was developed to further compare both groups of interest. A p-value of less than 0.05 was considered statistically significant.

**Results:**

A total of 2,357 patients were included, 1,704 of them allocated in the statin group and 653 in the non-statin group. After adjustments, statin use in the first 24 hours was associated with a lower incidence of in-hospital hyperglycemia (adjusted OR=0.61, 95% CI 0.46-0.80; p < 0.001) and lower need for insulin therapy (adjusted OR = 0.56, 95% CI 0.41-0.76; p < 0.001). These associations remained similar in the propensity-score matching models, as well as after several sensitivity analyses, such as after excluding patients who developed cardiogenic shock, severe infection or who died during index-hospitalization.

**Conclusions:**

Among statin-naïve patients admitted with ACS, early statin therapy was independently associated with lower incidence of in-hospital hyperglycemia. (Arq Bras Cardiol. 2021; 116(2):285-294)

## Introduction

There is established evidence that statins improve cardiovascular outcomes among patients with stable coronary artery disease (CAD).^1,2^ At the same time, patients with increased risk of CAD but without overt atherosclerosis may also derive benefit,^3,4^ making guidelines to recommend statins for those two groups.^5^ Also, statins play an important role in acute coronary syndromes (ACS)^6,7^and, in patients submitted to percutaneous revascularization, early therapy can confer additional benefit.^8,9^ Despite these benefits, concerns have been raised about increased risk of new onset diabetes mellitus (DM) with long-term statin therapy.^10-12^ Furthermore, statins can worsen glucose control in patients with known diagnosis of DM, or anticipate progression to overt DM in those with metabolic syndrome, impaired fasting blood glucose and glucose intolerance.^13,14^ Many possible mechanisms have been proposed to explain the influence of statins over glycemia.^15^ Statins could impair beta-cell function and decrease insulin secretion, a pathway directly related to inhibition of 3-hydroxy-3-methylglutaryl-coenzyme A (HMG-CoA) reductase or to other potential intracellular mechanisms.^16,17^ On the other hand, there is also some evidence that statins could decrease insulin resistance, thus compensating for the harmful mechanism mentioned before.^18^

While there is a huge body of data about long-term influence of statins over glycemia in stable CAD patients, there are scarce data in individuals with ACS. Despite the aforementioned concerns with impaired glucose tolerance in the long-term, due to their anti-inflammatory effects,^19,20^ statins could potentially decrease inflammation in the acute phase of ACS and thus indirectly reduce blood glucose levels related to the acute phase stress. Therefore, we hypothesized that, in patients hospitalized with ACS, early statin therapy would be associated with lower incidence of hyperglycemia during hospitalization in the coronary care unit (CCU).

## Methods

### Population and variables selection

We performed a retrospective data analysis of patients admitted with a diagnosis of ACS at the CCU of the Heart Institute of Sao Paulo University Medical School (*Instituto do Coração da Faculdade de Medicina da Universidade de São Paulo*). All consecutive patients admitted to our CCU with a diagnosis of ACS were prospectively registered in a dedicated database from January 1^st^, 1998 through May 1^st^, 2019. We identified statin-naïve patients at hospital admission and compared patients who received statins in the first 24 hours of admission (and continued during the whole hospitalization) versus those who did not.

Variables concerning ACS type – ST-elevation myocardial infarction (STEMI), non-ST elevation myocardial infarction (NSTEMI), or unstable angina (UA) – baseline demographic characteristics, risk factors, past CAD history or procedures, concomitant medications and baseline laboratory results were also collected. Blood glucose was obtained by blood sample daily; the first blood glucose level (admission glucose level) and the highest blood glucose level during hospitalization (i.e., peak glucose level) were registered in the database for further analysis.

We defined an ACS case as any patient presenting with new-onset ischemic symptoms at rest or worsening exertional ischemic symptoms requiring urgent hospital admission within the first seven days of symptoms onset. Myocardial infarction (MI) was defined according to the current Universal Definition of MI during data collection. STEMI was defined as persistent ST elevation of at least 1 mm in two or more contiguous leads (except V2-V3, where at least 1.5 mm was required in women and men older than 40 years and at least 2 mm in men younger than 40 years) or new/presumably new left bundle branch block at admission electrocardiogram (ECG). Cases not fulfilling criteria for MI were classified as UA. Patients were excluded if they were taking any statin immediately prior to index-hospitalization or if they did not have information regarding blood glucose levels or statin therapy at index hospitalization.

The primary outcome of interest in our analysis was the occurrence of in-hospital hyperglycemia, defined as peak blood glucose level > 200 mg/dL at any time during hospitalization, and secondary outcomes were peak blood glucose > median value of the peak blood glucose in our sample, and hyperglycemia requiring intravenous insulin therapy. This cut-off of 200 mg/dL was based on the most recent guidelines considering the suggested target level for glucose control in patients with ACS.^21^ We also explored the associations between hyperglycemia and in-hospital mortality.

### Laboratory routine

Every patient admitted with ACS had blood drawn from a forearm vein by venipuncture during admission. Blood was then centrifugated and sent to the laboratory, where glucose was determined by a standard procedure. For this first sample, fasting was not required, since we were interested in the first random blood glucose after ACS presentation. For further measures, fasting blood glucose was obtained, together with other routine laboratory samples.

### Statistical analysis

Categorical variables were compared with χ2 or Fisher’s exact tests as appropriate and were described as absolute numbers and percentages. Continuous variables are described as means ± standard deviations or median with interquartile ranges (IQR) and were compared with two-sample Student’s t-test (normal distribution) or the Mann-Whitney test (non-Gaussian distribution), as appropriate. Shapiro-Wilk test and visual analysis of histograms were used for normality testing.

Specifically, the Mann-Whitney test was used in the unadjusted analyses to compare the continuous outcomes of blood glucose between the two groups of interest. For the unadjusted analyses regarding the binary outcomes (incidence of hyperglycemia and in-hospital death), univariate logistic regression models were used.

In order to adjust for confounders, multivariable regression models were used in the adjusted analyses. Log-transformed blood glucose levels for the admission and peak glycemia were included in a multiple linear regression model. Transformation was done to meet normality of residuals assumption in the model. The model included as independent variables the baseline demographic data and comorbidity-related variables. A stepwise selection procedure was used to fit the model, with a threshold p-value of 0.2 to remove covariates and 0.05 for to add covariates in the model. A logistic regression model was also built following the same steps to assess hyperglycemia as a categorical variable (considering the three aforementioned definitions) and in-hospital death. Those models were adjusted for the following covariates: age, race, sex, DM, hypertension, hypercholesterolemia, smoking, heart failure (HF), prior MI, prior percutaneous coronary intervention (PCI), prior coronary artery bypass graft (CABG), prior stroke, creatinine clearance (CrCl) < 60 mL/min, ACS phenotype (STEMI versus NSTEMI or UA), Killip class 2 or more, year of admission (before or after 2010, which was the midpoint of the time span of the database), type of health insurance coverage (private versus government-funded), and GRACE (Global Registry of Acute Coronary Events) score.^22^ Values of glycated hemoglobin (HbA1c) and body mass index (BMI) were not available for all patients and were not included in the main model, but were included in sensitivity analyses (see below).

Additionally, a propensity score matching model was developed, considering the probability of receiving statin in the first 24 hours after admission. The model was built from a logistic regression and used a nearest neighbor of 1 and a caliper of 0.001, using the same variables used in the regression models. After matching, baseline variables were checked between the two groups to verify whether there remained imbalances, with both a p-value above 0.10 and a standardized mean difference less than 10% considered as appropriate, according to previous literature on the topic.^23^

For sensitivity analysis, we also ran additional models adjusting for all baseline variables, and for the use of aspirin, angiotensin converting-enzyme inhibitors (ACEI), angiotensin receptor blockers (ARB), oral betablockers, P2Y_12_ inhibitor, unfractionated heparin, low-molecular-weight heparin, intravenous betablockers, nitrates, and intravenous glycoprotein IIbIIIa inhibitors in the first 24 hours of admission. Besides that, we also ran a model including BMI in kg/m^2^ and another one including HbA1c at hospital admission as covariates. Finally, we also ran models excluding patients who developed cardiogenic shock, severe infection or who have died during the index hospitalization.

No imputation was used for missing data. Only individuals with valid information regarding statin use and blood glucose levels were included. All tests are two-tailed. A p-value of less than 0.05 was considered statistically significant. The software Stata^TM^ version 15.1 (Statacorp, College Station, TX, USA) was used for statistical calculations.

## Results

### Descriptive analyses

Out of 7,099 patients included in the database between January 1^st^ 1998 through May 1^st^ 2019, a total of 2,357 statin-naïve patients were included in this analysis – 1,704 were administered statin in the first 24 hours of hospital admission and 653 not (Figure 1). In the overall study population, the mean age was 62.9 ± 12.6 years, 713 patients (30.3%) were female, 789 (33.5%) had a history of known DM at admission, and 1,073 (45.5%) had STEMI as the clinical ACS presentation (Table 1).

**Figure 1:** – Flowchart of patients’ selection and allocation process.

**Table 1:** – Baseline characteristics, laboratory values, medications in the first 24 hours and revascularization strategies for the index-event according to study groups

Variables	Total (N = 2357)	Statin group (N=1704)	No statin group (N=653)	p-value
White race	2102 (89.2)	1500 (88.0)	602 (92.2)	0.004
Female sex	713 (30.3)	522 (30.6)	191 (29.3)	0.51
Age in yrs; mean ± SD	62.9 ± 12.6	62.6 ± 12.5	63.8 ± 12.9	0.038
Diabetes	789 (33.5)	591 (34.7)	198 (30.3)	0.045
Hypertension	1688 (71.6)	1246 (73.1)	442 (67.7)	0.009
Dyslipidemia	1241 (52.7)	912 (53.5)	329 (50.4)	0.17
Smoking	611 (25.9)	450 (26.4)	161 (24.7)	0.39
Prior MI	653 (27.7)	461 (27.1)	192 (29.4)	0.25
Prior CABG	327 (13.9)	234 (13.7)	93 (14.2)	0.75
Prior PCI	413 (17.5)	304 (17.8)	109 (16.7)	0.51
Prior stroke	113 (4.8)	87 (5.1)	26 (4.0)	0.25
Prior HF	212 (9.0)	162 (9.5)	50 (7.7)	0.16
CrCl ≤ 60 mL/min	1316 (55.8)	1053 (61.8)	263 (40.3)	< 0.001
STEMI as index-event	1073 (45.5)	781 (45.8)	292 (44.7)	0.63
Killip class 2 or more	447 (19.0)	308 (18.1)	139 (21.3)	0.075
GRACE score, mean ± SD	141.6 ± 47.5	140.5 ± 46.7	144.3 ± 49.4	0.14
Public health insurance	1736 (73.7)	1334 (78.3)	402 (61.6)	< 0.001
Included after jan-2010	950 (40.3)	870 (51.0)	80 (12.2)	< 0.001
BMI in kg/m^2^, median (IQR)^1 ^	25.7 (23.4 – 28.7)	25.7 (23.3 – 28.6)	25.9 (25.0 – 29.1)	0.19
Total cholesterol in mg/dL, median (IQR)^2^	182 (151-215)	182 (150-217)	178 (152-213)	0.51
LDL cholesterol in mg/dL; median (IQR)^2^	113 (87 – 143)	114 (86 – 144)	113 (90- 141)	0.96
Triglycerides in mg/dL; median (IQR)^2^	128 (91-180)	129 (92-184)	122 (89-171)	0.042
HDL cholesterol in mg/dL; median (IQR)^2^	37 (31-44)	37 (31-44)	38 (31-45)	0.36
HbA1c in %; median (IQR)^3^	5.9 (5.6 – 6.8)	5.9 (5.6 – 6.8)	5.9 (5.3 – 6.5)	0.054
Aspirin	2247 (95.4)	1646 (96.7)	601 (92.0)	< 0.001
P2Y12 inhibitor^4^	1199 (50.9)	1024 (60.1)	175 (26.8)	< 0.001
Oral beta-blocker	1424 (60.4)	1048 (61.5)	376 (57.6)	0.081
Intravenous beta-blocker	189 (8.0)	95 (5.6)	94 (14.4)	< 0.001
Nitrate	1461 (62.0)	1002 (58.8)	459 (70.3)	< 0.001
LMWH	1326 (56.3)	1099 (64.5)	227 (34.8)	< 0.001
UFH	772 (32.8)	463 (27.2)	309 (47.3)	< 0.001
ACEI/ARB	1651 (70.1)	1227 (72.0)	424 (64.9)	0.001
GpIIbIIIa inhibitor	867 (36.8)	632 (37.1)	235 (36.0)	0.62
Primary PCI	544 (23.1)	409 (24.0)	135 (20.7)	0.086
Fibrinolytic	264 (11.2)	195 (11.4)	69 (10.6)	0.55
**Revascularization for the index event^5^**				
PCI	1341 (56.9)	986 (57.9)	355 (54.5)	0.13
CABG	423 (18.0)	285 (16.7)	138 (21.1)	0.013
Medical management	637 (27.0)	460 (27.0)	177 (27.1)	0.96

Data are for n and % unless otherwise specified; 1- information about BMI was available for 287 patients; 2- information about cholesterol panel was available for 2062 patients; 3- information about HbA1c was available for 540 patients; 4- Seven patients, all in the statin group, were taking ticagrelor within the first 24 hours of admission, and all remaining patients on a P2Y_12 _inhibitor were taking clopidogrel; 5- CABG and PCI are not necessarily mutually exclusive since some patients may have been submitted to both. ACEI: angiotensin converting enzyme inhibitor; ARB: angiotensin receptor blocker; CABG: coronary artery bypass grafting; CrCl: creatinine clearance; GpIIbIIIa: glycoprotein IIbIIIa; GRACE: Global Registry of Acute Coronary Events; HF: heart failure; IQR: inter-quartile range; LMWH: low molecular weight heparin; MI: myocardial infarction; PCI: percutaneous coronary intervention; STEMI: ST-elevation myocardial infarction; UFH: unfractionated heparin.

As expected, there were several differences between patients taking statins compared with those not taking statins in the first 24 hours of hospitalization. Patients taking statins were younger, and more likely to have a history of hypertension, DM and CrCl < 60 mL/min at admission, among other differences. Also, they were more likely to have been included in the database after January 2010. Conversely, they were less likely to be white and to have private health insurance (Table 1). Patients taking statins in the first 24 hours were also more likely to be treated with aspirin, P2Y_12_ inhibitor and ACEI or ARB in the first 24 hours (Table 1). In terms of baseline laboratory values, patients taking statins in the first 24 hours had higher levels of triglycerides and a trend for higher levels of HbA1c, but similar levels of total cholesterol, LDL-cholesterol, and HDL-cholesterol (Table 1).

### Association between statin use in the first 24 hours and blood glucose levels

In the unadjusted analysis, blood glucose levels at admission were not different between patients taking statins and those not taking statins in the first 24 hours. However, patients taking statins in the first 24 hours, as compared to those not taking, had lower peak blood glucose (Table 2A).

In the adjusted multivariable analysis, statin therapy in the first 24 hours remained independently associated with lower peak blood glucose during index hospitalization (adjusted geometric means 139.0 versus 150.3 mg/dL, respectively; 95% CI of the difference of -15.9 to -6.5 mg/dL, adjusted p < 0.001 ). After adjustments, there remained no significant differences in admission blood glucose (Table 2B).

**Table 2B:** – Multivariable analysis2: adjusted geometric means of the blood glucose level according to statin treatment group

	Statin group (N=1704)	No statin group (N=653)	95% CI of the difference	Adjusted p-value
1^st^ blood glucose in mg/dL	124.4	125.2	- 5.2 to 3.3	0.64
Peak blood glucose in mg/dL	139.0	150.3	-15.9 to -6.5	< 0.001

Adjusted for age, race, sex, diabetes, hypertension, hypercholesterolemia, smoking, heart failure, prior myocardial infarction, prior percutaneous coronary intervention, prior coronary artery bypass graft, prior stroke, creatinine clearance < 60 ml/min, ACS phenotype (STEMI versus NSTEMI), Killip class 2 or more, GRACE score, health insurance coverage status and period of inclusion (before versus after January 2010) 1- Unadjusted analysis made by Mann Whitney test. 2- Multivariable analysis performed by multivariate linear regression

In a propensity score matched analysis, 500 patients from the statin group were matched with a similar number of patients from the group without statin therapy. After matching, baseline characteristics used to build the model were well balanced between the two groups, without any p-value < 0.10 nor any standardized mean difference > 10 % (Supplementary Table 1 and Supplementary Figure 1). Considering the propensity score matched analysis, statin therapy remained significantly associated with lower levels of peak blood glucose (Supplementary Table 2).

**Table 2A:** – Unadjusted analysis1: median values of the first blood glucose level and peak blood glucose according to statin treatment groups

	Statin group (N=1704)	No statin group (N=653)	P-value
First blood glucose level (mg/dL); median (IQR)	116 (97 – 159)	113 (95 – 153)	0.22
Peak blood glucose (mg/dL); median (IQR)	124 (101 – 175)	134 (106 – 196)	< 0.001

### Association between statin and occurrence of in-hospital hyperglycemia

In the unadjusted analysis, statin therapy in the first 24 hours was associated with lower incidence of hyperglycemia, including peak blood glucose above 200 mg/dL, peak blood glucose above the median, and hyperglycemia with the need for insulin therapy (Supplementary Table 3).

**Table 3:** – Associations between blood glucose levels and in-hospital death

	Unadjusted OR (95% CI); p-value	Adjusted OR (95% CI); p-value
Admission blood glucose (for every 10 mg/dL)	1.03 (1.01 – 1.06); < 0.001	1.02 (0.99 – 1.04); 0.12
Peak blood glucose (for every 10 mg/dL)	1.06 (1.04 – 1.08); < 0.001	1.05 (1.03 – 1.07); < 0.001
Admission blood glucose > 200 mg/dL	1.72 (1.15 – 2.56); 0.008	1.42 (0.90-2.24); 0.14
Peak blood glucose > 200 mg/dL	3.06 (2.20-4.28); <0.001	2.70 (1.76-4.16); <0.001

Adjusted for: age, race, sex, diabetes, hypertension, hypercholesterolemia, smoking, heart failure, prior myocardial infarction, prior percutaneous coronary intervention, prior coronary artery bypass graft, prior stroke, creatinine clearance < 60 ml/min, ACS phenotype (STEMI versus NSTEMI), Killip class 2 or more, GRACE score, type of health insurance and period of inclusion (before versus after January 2010). Models were performed using univariate logistic regression for unadjusted analyses and multivariate logistic regression for adjusted analyses

After adjusted multivariable analysis, statin therapy remained independently associated with lower incidence of peak blood glucose above 200 mg/dL (adjusted OR = 0.61, 95% CI 0.46-0.80; p < 0.001), as well as peak blood glucose above the median and hyperglycemia requiring insulin therapy (See Supplementary Table 3 and Figure 2A for more details).

**Figure 2:** Adjusted risk of in-hospital hyperglycemia according to different definitions. A Adjusted by multivariable logistic regression. B: adjustment by propensity score matching. Adjusted for: age, race, sex, diabetes, hypertension, hypercholesterolemia, smoking, heart failure, prior myocardial infarction, prior percutaneous coronary intervention, prior coronary artery bypass graft, prior stroke, creatinine clearance < 60 ml/min, ACS phenotype (STEMI versus NSTEMI), Killip class 2 or more, GRACE score, type of health insurance and period of inclusion (before versus after Jan-2010).

In the propensity score matching analysis, results were similar to those obtained in the multivariable regression approach, with significant associations between statin use and lower risk of hyperglycemia according to all three definitions (Supplementary Table 3 and Figure 2B).

### Sensitivity analyses

Associations between statin therapy and lower incidence of hyperglycemia (peak blood glucose > 200 mg/dL) remained consistent after several sensitivity analyses were conducted, such as including other concomitant medications in the model. Results for the primary endpoint also remained consistent in a model that considered the date of inclusion in the database as a continuous variable. In another analysis, where patients were stratified according to period of inclusion (before versus after January 2010), there was no significant effect modification for the primary endpoint Additionally, when patients who developed cardiogenic shock or severe infection, or who did not survive up to hospital discharge were excluded from the analysis, there remained significant associations between use of statin in the first 24 hours and lower occurrence of hyperglycemia. Finally, in models that included HbA1c (%) or BMI as covariates, although the point estimates for the OR´s remained similar, no significant association remained, probably due to the small number of patients with those two variables available. These results are described in Supplementary Tables 4 to 11.

### Association between blood glucose levels and in-hospital mortality

In the overall study population, higher peak blood glucose levels were independently associated with higher in-hospital mortality (OR 1.05; 95%CI 1.03-1.07 for every 10 mg/dL; p < 0.001). On the other hand, admission blood glucose was not independently associated with in-hospital mortality (Table 3).

## Discussion

### Study main findings

There are several important results of this study. First, that statin use in the first 24 hours of hospitalization in ACS was highly associated with comorbidities and, more importantly, there was an important temporal association, such that, in our data, a large difference existed between patients included before versus patients included after 2010. This is likely related to the body of evidence accumulated regarding the use of statins in ACS,^6,7^ although there is no guideline recommendation supporting initiation of statin therapy within the first 24 hours of hospital admission.^5,21^ Second, there was an independent association between early statin therapy and lower incidence of in-hospital hyperglycemia. This association was observed in two different adjusted models (logistic regression and propensity score matching) and after several sensitivity analyses performed to verify the consistency of the findings. Therefore, despite the established risk of new-onset DM in the chronic phase, our results probably exclude any apparent harm, or impairment in glucose tolerance with statins during the acute phase of ACS, a period where the surge of catecholamines and inflammatory mediators may increase susceptibility to stress hyperglycemia and its potential clinical consequences.^24^

### Comparison with previous studies

Despite the vast literature investigating the effects of statins over impaired glucose tolerance chronically, few reports have investigated any possible effect in the acute scenario. Yan et al.^25^ have reported a higher risk of stress hyperglycemia in patients with acute MI receiving statins, but the lack of adjusted analysis and the arbitrary cut-off for stress hyperglycemia weaken the conclusions of that study. Sposito et al.^26^ studied this issue in patients hospitalized with STEMI, showing that simvastatin 80 mg decreased insulin sensitivity compared to simvastatin 10 mg assessed by euglycemic hyperinsulinemic clamp.^26^ Although these results appear to contrast to ours, the inclusion of only patients without DM and the use of euglycemic hyperinsulinemic clamp limit the generalization of their finds to a real-world scenario such as our study. Nevertheless, it is possible that, despite an adverse effect over insulin resistance early in the acute phase of ACS, statins could compensate for that by reduction in inflammatory response, leading to a decrease in glucose blood levels.

The association between statins and lower blood glucose in stress situations could be at least partially explained by the direct effects of statins on inflammation, which is well-established in the literature.^19^ Other reports have suggested that those pleiotropic effects from statins may occur early in the course of ACS. Two randomized studies have demonstrated that short-duration (less than 5 days) treatment with rosuvastatin, compared to placebo, decreased the incidence of post-contrast acute kidney injury.^27,28^ This effect seemed to be mediated by an anti-inflammatory action, since no lipid-lowering effect would be expected in such a short timeframe.^29^ Additionally, an early statin loading has been found to decrease the incidence of peri-procedural MI in one meta-analysis.^8^ However, a larger randomized study, the SECURE-PCI trial, did not find a decrease in ischemic events after ACS with an 80 mg loading dose of atorvastatin,^9^ despite a potential benefit in the subgroup of patients who were submitted to PCI after randomization.^30^

### Hyperglycemia and mortality after ACS

The impact of hyperglycemia on survival after ACS is well-established both in patients with and without DM.^24^ In a sub-analysis in the CARDINAL trial, Goyal et al.^31^ have suggested that persistence of elevated blood glucose through 24 hours after admission was even more associated with lower survival than admission high blood glucose.^31^ While there is an association between glycemia and mortality, it is not completely understood whether high blood glucose is a direct mediator of increased cell death and injury during MI, or just a marker of increased baseline risk. From a biological perspective, hyperglycemia may be associated with direct impairment of microcirculation and adverse left ventricular remodeling.^32^ On the other hand, findings from randomized studies that failed to demonstrate better prognosis with stricter glycemic control support the latter hypothesis.^33,34^ Nevertheless, if high blood glucose could be partially implicated in myocardial damage during MI, our results are reassuring, since they likely exclude a harmful effect of statins on glucose metabolism in the acute phase of ACS.

### Study limitations

Our study has several limitations. First, we did not collect detailed information on dosages and types of statins that were used. Although some studies have found differential effects among different statins on glucose metabolism, other studies have suggested that the risk of DM with statins might be a class effect.^35,36^ Second, our database spans over a long timeline, including patients since 1998, when the use of statins during acute phase of MI was less widespread. However, we considered this covariate in the adjusted models and in additional sensitivity analyses, thus supporting the findings that the association was not spuriously driven by this confounding factor. Third, we did not collect detailed information about indications and contraindications for starting or not early statin therapy in ACS. Accordingly, it might be possible that patients at a higher risk of death or in critical clinical condition were less likely to be administered statins in the first 24 hours by the treating physician. Several factors, in addition to the drug effect on glucose metabolism, may have influenced the decision to initiate statins in the first 24 hours. Also, although adjustments were made for several comorbidities and other demographic and socioeconomic factors, unknown residual confounders may remain. However, the GRACE score, a well-established predictor of in-hospital death among patients with ACS^22^ was included as covariate, and we also performed several sensitivity analyses excluding patients who developed cardiogenic shock or severe infection, and those who died during index hospitalization. Fourth, due to the retrospective nature of our analysis, it could not be determined whether our findings were subject to reporting bias. Fifth, our data come from a single-center database, so it is uncertain whether our findings could be extrapolated to other countries or to different standards of practice in other hospitals. Finally, due to the observational nature of our study, we cannot make any causal inference but only conclude about associations, so that our findings are only hypothesis-generating and should be confirmed in dedicated randomized trials.

## Conclusion

In patients admitted with ACS, statin therapy in the first 24 hours was associated with lower incidence of in-hospital hyperglycemia. This finding suggests that, while statins can increase the risk of new-onset DM in the long-term, they could be associated with salutary effects over glucose metabolism in the short-term in ACS.
